# Notch3-Mediated mTOR Signaling Pathway Is Involved in High Glucose-Induced Autophagy in Bovine Kidney Epithelial Cells

**DOI:** 10.3390/molecules27103121

**Published:** 2022-05-13

**Authors:** Yaocheng Cui, Jing Fang, Hongrui Guo, Hengmin Cui, Junliang Deng, Shumin Yu, Liping Gou, Fengyuan Wang, Xiaoping Ma, Zhihua Ren, Yue Xie, Yi Geng, Ya Wang, Zhicai Zuo

**Affiliations:** 1 Key Laboratory of Animal Disease and Human Health of Sichuan Province, College of Veterinary Medicine, Sichuan Agricultural University, Chengdu 611130, China; cuiyaocheng@stu.sicau.edu.cn (Y.C.); fangjing4109@163.com (J.F.); guohongrui@sicau.edu.cn (H.G.); cuihengmin2008@sina.com (H.C.); dengjl213@126.com (J.D.); yayushumin@163.com (S.Y.); glping0827@163.com (L.G.); mxp886@sina.com (X.M.); zhihua_ren@126.com (Z.R.); zhandegaokandey123@163.com (Y.X.); gengyisicau@126.com (Y.G.); wangyayang@126.com (Y.W.); 2 College of Animal & Veterinary Sciences, Southwest Minzu University, Chengdu 610041, China; wfy_sccd@163.com

**Keywords:** autophagy, high glucose, mTOR, MDBK cells, Notch3

## Abstract

It is reported that Notch3 and mTOR signaling pathways are involved in autophagy, and both can be activated by high glucose (HG). However, the relationship between Notch3 and mTOR and how Notch3 affects mTOR to regulate HG-induced autophagy in bovine kidney epithelial cells is still unclear. The purpose of this study is to explore how Notch3 affects mTOR to modulate HG-induced autophagy in bovine kidney cells. Our results showed that HG treatment significantly decreased the cell viability of MDBK cells in a dose-dependent manner. HG treatment significantly increased the expression of LC3-II/I ratio and Beclin1 protein and significantly decreased the expression of p62 protein. Consistently, LC3 fluorescence signal formation was detected by immunofluorescence in both dose and time-dependent manners. In addition, HG treatment significantly increased the expression of Notch3 protein and decreased the expression of the p-mTOR protein in both dose and time-dependent manners. Inhibition of Notch3 upregulated the expression of p-mTOR and p62 protein, and downregulated the expression of LC3-II/I ratio and Beclin1 protein. Besides, the function of Notch3 was investigated. In this study, inhibition of Notch3 activity significantly increased the viability of HG-stimulated MDBK cells. In summary, our results revealed that the Notch3-mediated mTOR signaling pathway was involved in HG-induced autophagy in MDBK cells.

## 1. Introduction

Hyperglycemia can cause serious diabetic kidney disease or stress hyperglycemia in humans, mice, and rats [1,2,3]. The concentrations of glycemia in beef cattle significantly increase during transportation stress [4,5,6]. However, the cytological molecular mechanism of hyperglycemia in beef cattle injury remains unclear. The kidney is one of the organs most susceptible to glucose concentration [7]. HG is commonly used to simulate hyperglycemia in in vitro experiments. Previous studies usually focus on inflammation, apoptosis, and pyroptosis in HG-induced renal cells [8,9]. Moreover, studies have found that autophagy plays an important role in HG-induced cell injury [10] and can regulate inflammation, apoptosis, and pyroptosis [11,12,13]. For example, autophagy was activated by p53 deacetylation in diabetic mice and HG-treated HK-2 cells [14].

Notch receptors are the most conserved signaling molecules that are widely distributed on the surface of a variety of cells [15]. In mammals, there are four Notch receptors Notch1, Notch2, Notch3 and Notch4 [16]. The abnormal activation of Notch3 plays a key role in kidney disease and directly affects the prognosis of kidney disease [17,18,19]. mTOR signaling, one of the main signaling pathways regulating autophagy, controls many aspects of the autophagy process, such as initiation, process, and termination [20]. Previous studies have reported that mTOR is the major upstream regulator of Notch3 [21]. However, a recent study found that Notch3 protein expression was not affected when mTOR was inhibited, suggesting that Notch3 may be located upstream of mTOR [22]. To date, whether Notch3 has a regulatory effect on mTOR activity still remains unclear. Autophagy involves a lot of signaling pathways which makes it an extremely complex process to be regulated [23]. Notch3 and mTOR are reported to be highly expressed in high glucose-stimulated cells [24,25]. Notch3 has also been found to play an important regulatory role in autophagy [26,27,28]. Regrettably, it is still unclear whether Notch3/mTOR plays a regulatory role in HG-induced autophagy. 

In this study, we explored how Notch3 affected mTOR to regulate HG-induced autophagy in bovine kidney cells. The present study may provide a certain molecular basis for the pathogenesis of hyperglycemia-induced nephropathy.

## 2. Results

### 2.1. HG Treatment Induced Autophagy Markers in MDBK Cells

**Figure 1:**
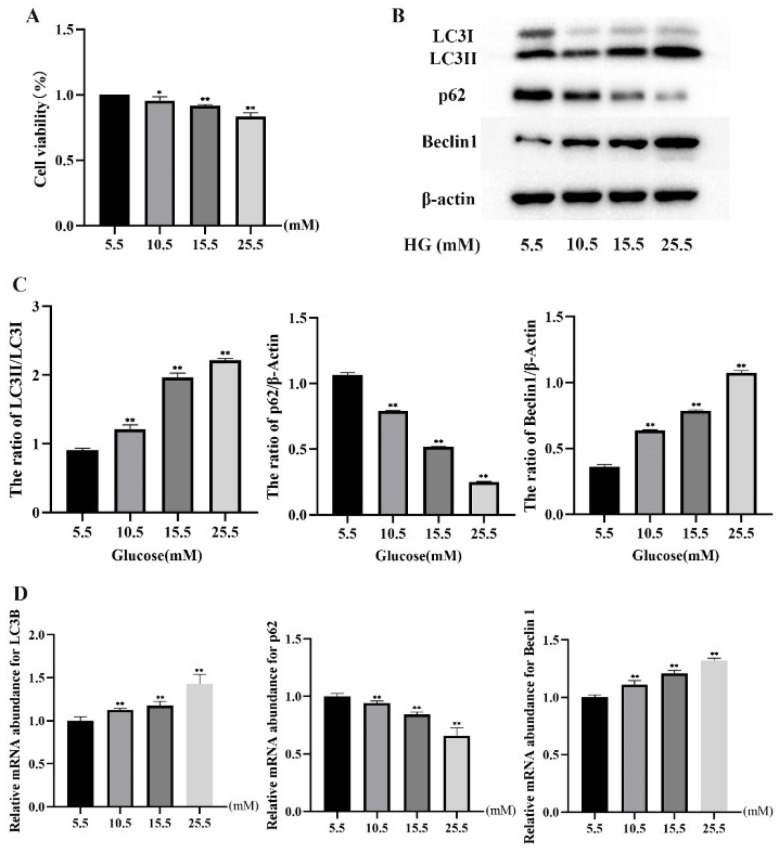
Effects of different concentrations of glucose on autophagy in MDBK cells. MDBK cells were treated with 5.5, 10.5, 15.5, and 25.5 mM glucose for 24 h, respectively. Western blot, qRT-PCR, and CCK-8 assay were used to assess protein expression, gene expression, and cell viability, respectively. (**A**) The cell viability; (**B**,**C**) The protein level of LC3-II/I, p62, and Beclin1; (**D**) The mRNA expression level of LC3B, p62, and Beclin1. * *p* < 0.05 and ** *p* < 0.01 compared with control group.

To evaluate the effect of HG on MDBK cells viability, the cells were treated with 5.5, 10.5, 15.5, and 25.5 mM glucose for 24 h, and the cell proliferation was examined. The results showed that the MDBK cells viability of the 10.5, 15.5, and 25.5 mM glucose treatment groups decreased significantly (*p* < 0.05) compared with the 5.5 mM glucose treatment groups, respectively (Figure 1A). Based on the above observations, glucose concentrations (5.5, 10.5, 15.5, and 25.5 mM) were chosen for subsequent experiments.

**Figure 2:**
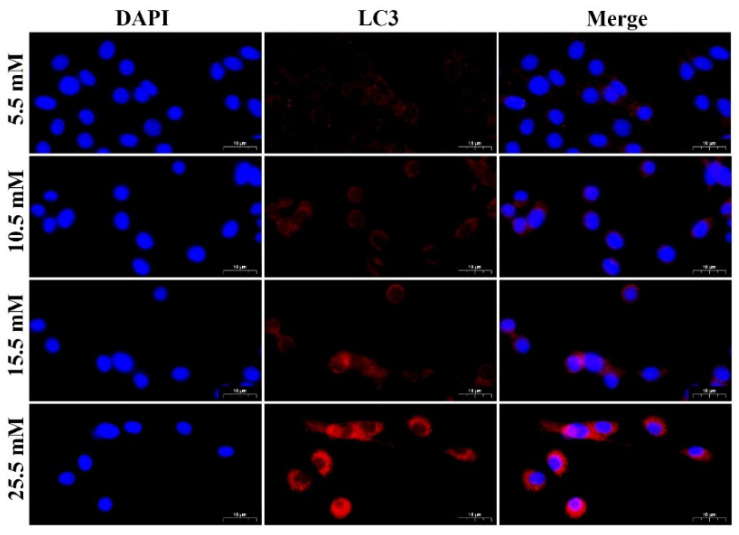
The fluorescence distribution of LC3 in MDBK cells after different concentrations of glucose stimulation. MDBK cells were treated with 5.5, 10.5, 15.5, and 25.5 mM glucose for 24 h, respectively. LC3 was labeled with red fluorescence by Cy3, and the nucleus was labeled with blue fluorescence by DAPI. Scale bar, 10 µm.

Next, to explore the effect of different concentrations of glucose (5.5, 10.5, 15.5, and 25.5 mM) on autophagy in MDBK cells, the protein and mRNA expression levels of LC3, p62, and Beclin1 in MDBK cells were detected by Western blot and qRT-PCR, and the intensity of red fluorescent labeled LC3 was detected by immunofluorescence assay. 10.5, 15.5 and 25.5 mM glucose treatment groups showed a significant increase (*p* < 0.01) in the protein levels of LC3-II/I and Beclin1 whereas the protein level of p62 was decreased significantly (*p* < 0.01) compared with 5.5 mM glucose treatment group (Figure 1B,C). Consistent with the protein experiment results, relative to the 5.5 mM glucose treatment groups (Figure 1D), the mRNA expression level of LC3B and Beclin1 was increased significantly (*p* < 0.01), and the mRNA expression level of p62 was decreased significantly (*p* < 0.01) in the 10.5, 15.5 and 25.5 mM glucose treatment groups. An immunofluorescence assay was used for measuring protein levels of LC3 in the MDBK cells in the present study. As shown in Figure 2, the 10.5, 15.5, and 25.5 mM glucose treatment groups showed a significant increase in the intensity of red fluorescent labeled LC3 compared with the 5.5 mM glucose treatment group. Moreover, the 25.5 mM glucose treatment group was the most significant among these groups.

**Figure 3:**
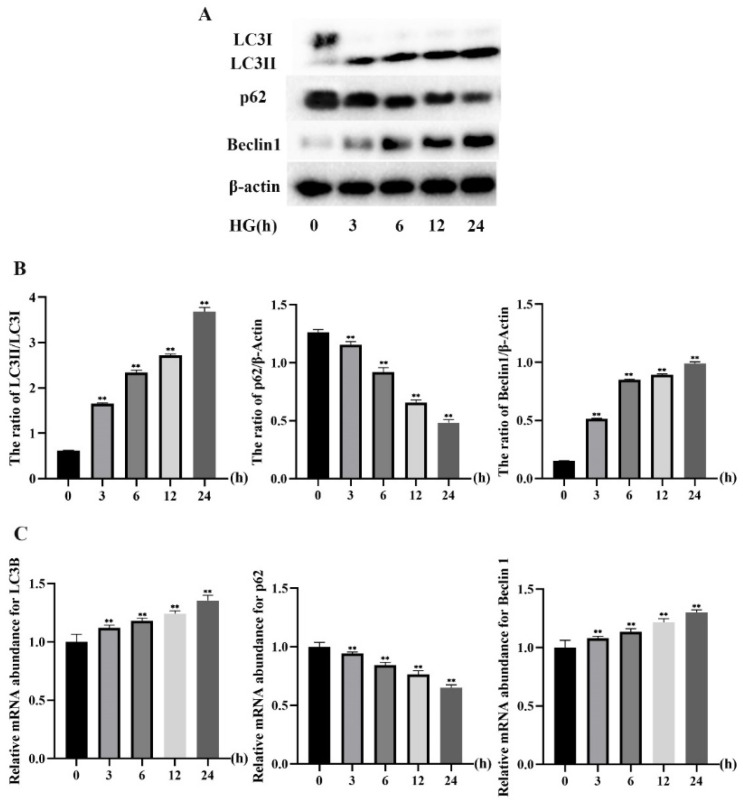
Effects of different treatment times of HG on autophagy in MDBK cells. MDBK cells were treated with 25.5 mM glucose for 0, 3, 6, 12, and 24 h, respectively. Western blot and qRT-PCR were used to assess protein expression, and gene expression, respectively. (**A**,**B**) The protein level of LC3-II/I, p62, and Beclin1; (**C**) The mRNA expression level of LC3B, p62, and Beclin1. ** *p* < 0.01 compared with control group.

**Figure 4:**
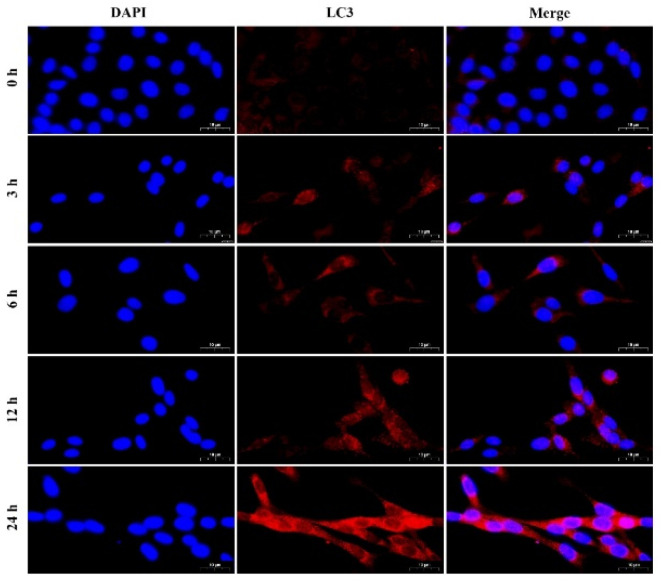
The fluorescence distribution of LC3 in MDBK cells after different treatment times of HG stimulation. MDBK cells were treated with 25.5 mM glucose for 0, 3, 6, 12, and 24 h, respectively. LC3 was labeled with red fluorescence by Cy3, and the nucleus was labeled with blue fluorescence by DAPI. Scale bar, 10 µm.

Furthermore, to explore the effect of different HG treatment times on autophagy, MDBK cells were treated with 25.5 mM glucose for 0, 3, 6, 12, and 24 h. The protein and mRNA expression levels of LC3, p62, and Beclin1 in MDBK cells were detected by Western blot and qRT-PCR, and the intensity of red fluorescent labeled LC3 was detected by immunofluorescence assay. Figure 3A, B illustrated that the 3, 6, 12 and 24 h treatment groups showed a significant increase in the protein levels of LC3-II/I and Beclin1 (*p* < 0.01) whereas the protein level of p62 was decreased significantly (*p* < 0.01) compared with 0 h treatment group. Consistent with the protein experiment results, relative to the 0 h treatment groups (Figure 3C), the mRNA expression level of LC3B and Beclin1 was increased significantly (*p* < 0.01), whereas the mRNA expression level of p62 was decreased significantly (*p* < 0.01) in the 3, 6, 12 and 24 h treatment groups. In addition, compared with other groups, the protein and mRNA expression levels of the 24 h treatment group were the most significant. As shown in Figure 4, the 3, 6, 12, and 24 h treatment groups showed a significant increase in the intensity of red fluorescent labeled LC3 compared with the 0 h treatment group. Moreover, the 24 h treatment group was the most significant among these groups.

To investigate whether HG induces autophagic flux in MDBK cells, chloroquine (CQ) was used. As shown in Figure S1 (Supplementary Materials), incubation of cells with CQ resulted in significantly increased LC3-II expression (*p* < 0.01), and treatment with HG and CQ revealed a further increase in LC3-II expression (*p* < 0.01).

In summary, these data indicated that HG induced autophagy in MDBK cells.

### 2.2. HG Induced Notch3 and mTOR Signaling Pathways in MDBK Cells

**Figure 5:**
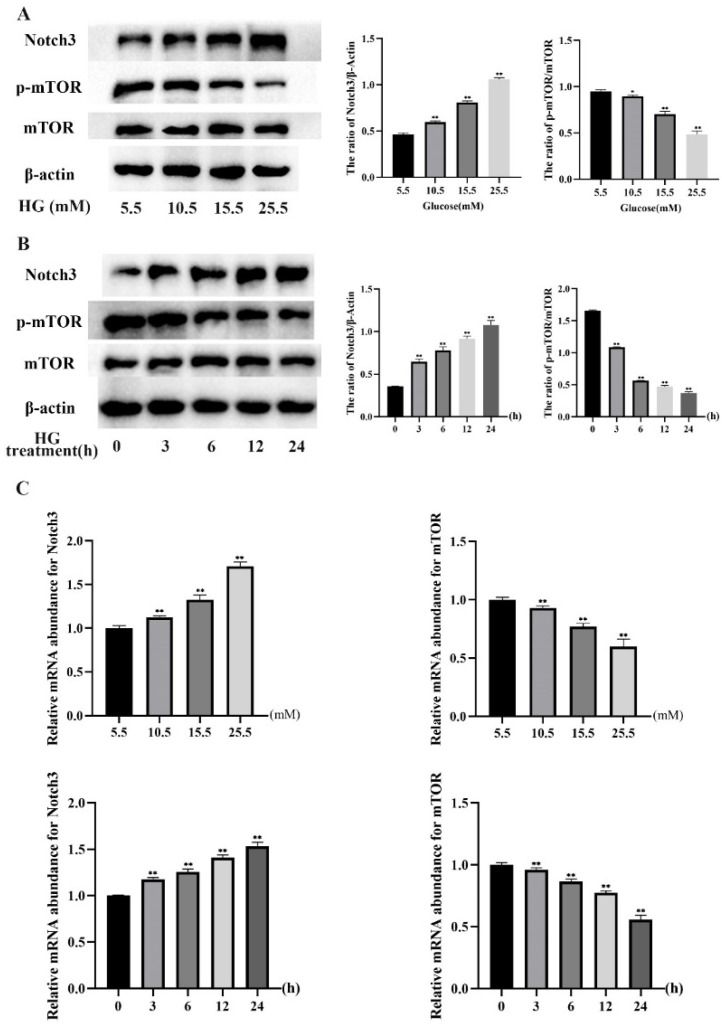
HG induced Notch3 and mTOR signaling pathways in MDBK cells. MDBK cells were treated with 5.5, 10.5, 15.5, and 25.5 mM glucose for 24 h, respectively; MDBK cells were treated with 25.5 mM glucose for 0, 3, 6, 12, and 24 h, respectively. Western blot, and qRT-PCR were used to assess protein expression, and gene expression, respectively. (**A**,**B**) The protein level of Notch3, mTOR, and p-mTOR; (**C**) The mRNA expression level of Notch3 and mTOR. * *p* < 0.05 and ** *p* < 0.01 compared with control group.

To investigate the role of Notch3 and mTOR in MDBK cells induced by HG, the protein and mRNA expression levels of Notch3 and mTOR in MDBK cells were detected by Western blot and qRT-PCR (Figure 5A–C). Figure 5A showed that the protein level of Notch3 was increased significantly (*p* < 0.01) in the 10.5, 15.5, and 25.5 mM glucose treatment groups compared with the 5.5 mM glucose treatment group. Meanwhile, the protein level of p-mTOR was decreased significantly (*p* < 0.05) in the 10.5, 15.5, and 25.5 mM glucose treatment groups compared with the 5.5 mM glucose treatment group. Consistent with the protein experiment results, relative to the 5.5 mM glucose treatment groups (Figure 5C), the mRNA expression level of Notch3 was increased significantly (*p* < 0.01), whereas the mRNA expression level of mTOR was decreased significantly (*p* < 0.01) in the 10.5, 15.5 and 25.5 mM glucose treatment groups. Moreover, compared with other groups, the protein and mRNA levels of Notch3 and p-mTOR were most significant in the 25.5 mM glucose treatment group.

Furthermore, the protein levels of Notch3 showed a significant increase (*p* < 0.01) at 3, 6, 12, and 24 h in the treatment groups compared with the 0 h treatment group (Figure 5B). The protein level of p-mTOR was decreased significantly (*p* < 0.01) in the 3, 6, 12, and 24 h treatment groups compared with the 0 h treatment group (Figure 5B). Similar to the protein experiment results, relative to the 0 h treatment groups (Figure 5C), the mRNA expression level of Notch3 increased significantly (*p* < 0.01), whereas the mRNA expression level of mTOR decreased significantly (*p* < 0.01) in treatment groups at 3, 6, 12 and 24 h of treatment. Moreover, the protein and mRNA levels of Notch3 and mTOR were the most significant in the treatment group at 24 h compared with other groups.

These results revealed that HG induced Notch3 and mTOR signaling pathways in MDBK cells.

### 2.3. Notch3-Mediated mTOR Signaling Pathway was Involved in HG-Induced Autophagy in MDBK Cells

**Figure 6:**
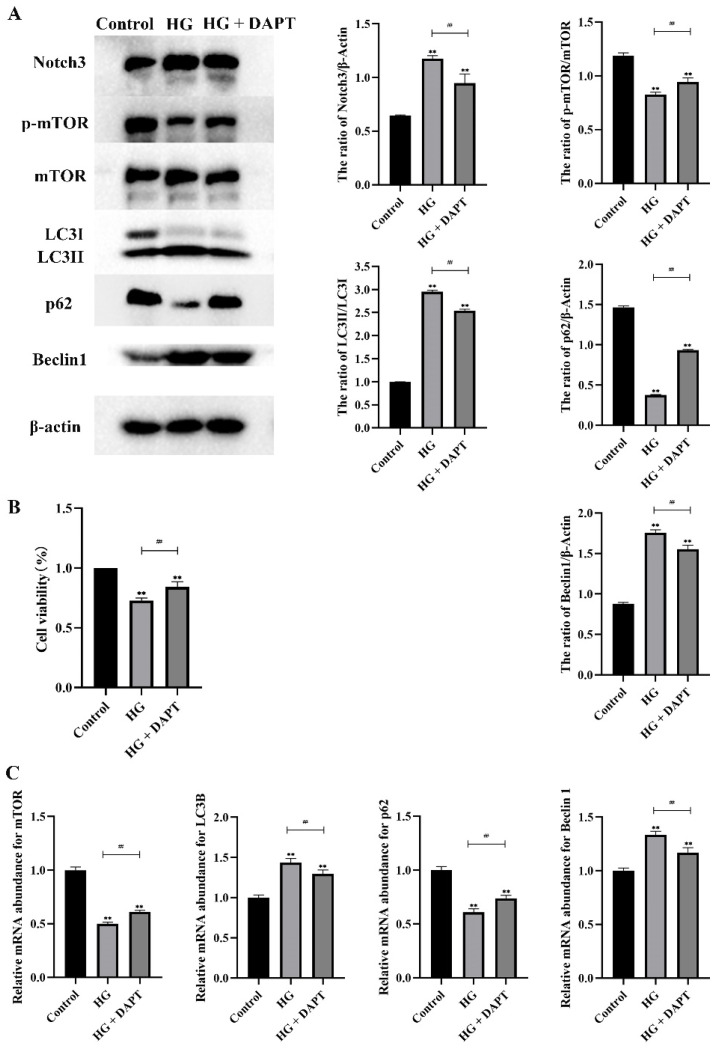
Notch3-mediated mTOR signaling pathway was involved in HG-induced autophagy in MDBK cells. MDBK cells are pretreated for 6 h with 10 μM DAPT, and then stimulated for another 24 h with 25.5 mM glucose. Western blot, CCK-8 assay, and qRT-PCR were used to assess protein expression, cell viability, and gene expression, respectively. (**A**) The protein level of Notch3, mTOR, p-mTOR, LC3-II/I, p62, and Beclin1; (**B**) The cell viability; (**C**) The mRNA expression level of Notch3, mTOR, LC3B, p62, and Beclin1. ** *p* < 0.01 compared with control group; ##: *p* < 0.01.

To investigate the relationship between Notch3 and mTOR in autophagy induced by HG, MDBK cells were pretreated with 10 μM DAPT, a specific Notch inhibitor. Firstly, western blot results showed that Notch3, LC3-II/I, and Beclin1 proteins were increased significantly (*p* < 0.01), whereas the p-mTOR and p62 proteins were decreased significantly (*p* < 0.01) in the HG treatment group compared with the control group (Figure 6A). Notch3, LC3-II/I, and Beclin1 proteins were decreased significantly (*p* < 0.01), whereas the p-mTOR and p62 proteins were increased significantly (*p* < 0.01) in the HG + DAPT treatment group compared with the HG treatment group (Figure 6A). As shown in Figure 6C, compared with the control group, the mRNA levels of LC3B and Beclin1 increased significantly (*p* < 0.01), whereas the mRNA levels of mTOR and p62 decreased significantly (*p* < 0.01) in the HG treatment group. The mRNA levels of LC3B and Beclin1 decreased significantly (*p* < 0.01), whereas the mRNA levels of mTOR and p62 increased significantly (*p* < 0.01) in the HG + DAPT treatment group compared with the HG treatment group.

Besides, MDBK cell viability was examined by CCK-8 assay in the control group (5.5 mM glucose treatment group), HG group (25.5 mM glucose treatment group), and HG + DAPT group (25.5 mM glucose + 10 μM DAPT treatment group). The results showed that the MDBK cell viability of the HG treatment groups decreased significantly (*p* < 0.05) compared with the control groups (Figure 6B). Moreover, compared with the HG group, the viability of the MDBK cells increased significantly in the HG + DAPT group (*p* < 0.01).

**Figure 7:**
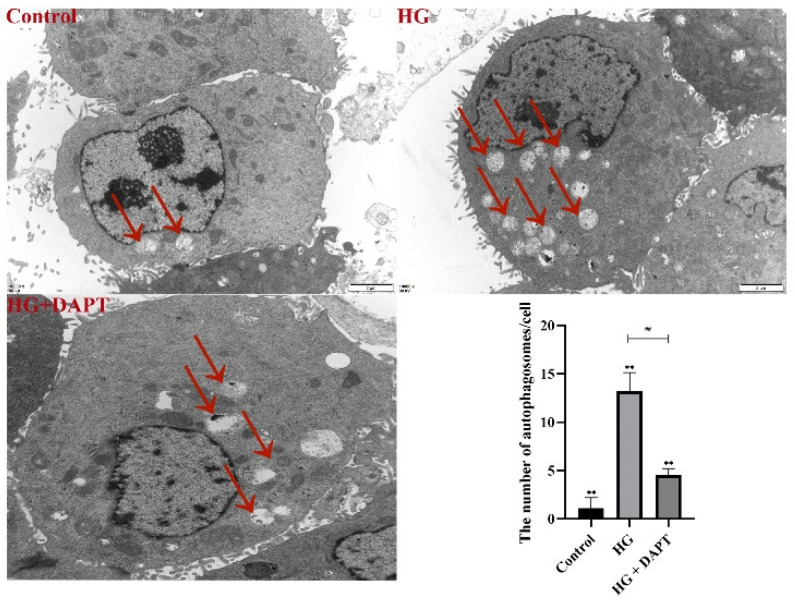
Autophagosomes under TEM. MDBK cells are pretreated for 6 h with 10 μM DAPT, and then stimulated for another 24 h with 25.5 mM glucose. TEM images of autophagic vacuoles (red double arrow) were shown in MDBK cells from Control, HG, and HG + DAPT treatment groups (Scale bar: 2 μm). ** *p* < 0.01 compared with control group; ##: *p* < 0.01.

Furthermore, the autophagosomes also were detected by TEM in the current study. The results showed that compared with the control treatment group, the formation of autophagosomes was increased in the HG treatment group (Figure 7). Moreover, compared with the HG group, the formation of autophagosomes was decreased in the HG + DAPT treatment group (Figure 7).

**Figure 8:**
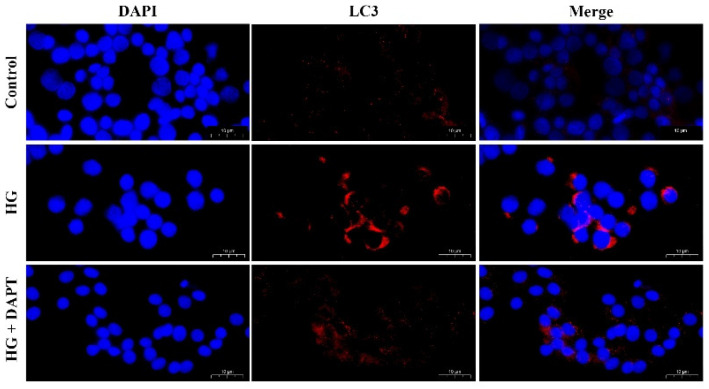
The fluorescence distribution of LC3. MDBK cells are pretreated for 6 h with 10 μM DAPT, and then stimulated for another 24 h with 25.5 mM glucose. LC3 was labeled with red fluorescence by Cy3, and the nucleus was labeled with blue fluorescence by DAPI. Scale bar, 10 µm.

Similar results were demonstrated in the immunofluorescence assay. As presented in Figure 8, compared with the control group, the intensity of red fluorescent labeled LC3 in the HG treatment group was increased. In addition, the intensity of red fluorescent labeled LC3 was decreased in the HG + DAPT treatment group compared with the HG treatment group.

In summary, these findings demonstrated that the Notch3-mediated mTOR signaling pathway was involved in HG-induced autophagy in MDBK cells.

## 3. Discussion

Notch3 and mTOR signaling pathways have been reported to be involved in autophagy, and both can be activated by HG. However, the relationship between Notch3 and mTOR and whether HG induces autophagy in renal cells by Notch3/mTOR signaling pathway are still unclear. In the present study, we investigated how Notch3 influenced mTOR to regulate HG-induced autophagy in bovine renal cells.

Autophagy is an important process for the cell survival and maintenance of cells where organelles, proteins, and macromolecules in the cytoplasm and the circulation of breakdown products are degraded [29]. Moreover, autophagy plays an important regulatory role in HG-induced renal cell injury [30]. The glucose concentration in vitro is generally 5.5 mM~25.5 mM, which is used to simulate normal blood glucose and hyperglycemia respectively [31,32]. Furthermore, blood glucose levels in beef cattle subjected to transport stress increased from 5.5 mM to 15.2 mM [5]. As a result, the glucose concentration range for this test was set at 5.5 to 25.5 mM. In most studies, HG-induced autophagy in renal cells occurred within 24 h [33,34]. For example, Chen et al. [33] found that when human proximal tubular epithelial cells were treated with HG for 24 h, the LC3 protein expression increased, and the p62 protein expression decreased. Therefore, the action time range of HG is set at 0~24 h. Firstly, we examined whether HG treatment induces autophagy in MDBK cells. Our results showed a significant increase of LC3-II/LC3-I and Beclin1 whereas a remarkable decrease in p62 in both dose and time-dependent manners. This indicated that HG induced autophagy in MDBK cells. These results are consistent with previous findings [31,32,33]. Several evolutionarily-conserved genes (called autophagy-related genes, such as LC3, Beclin1, and p62) are involved in the process of autophagy [35,36,37]. Meanwhile, immunofluorescence results showed that the intensity of fluorescence increased in MDBK cells after HG treatment.

Next, we investigated the mechanism of autophagy induced by HG. mTOR is a significant negative regulator of autophagy, conscientious for nutrient consumption, low energy, or oxidative stress [38]. In this experiment, we observed a reduction in p-mTOR, indicating that the HG was capable of inhibiting mTOR activities. Consistent with our results, Zhao et al. [39] have also proposed that HG exposure induces autophagy in H9c2 cells by inhibiting mTOR. It has been demonstrated that highly active Notch3 is generally found to be related to kidney injuries [40,41,42]. In this research, an increase in Notch3 implied that the Notch3 signaling pathway was involved in autophagy induced by HG in MDBK cells. Similar results were also observed in the study by Huang et al. [19], which reported that the expression of Notch3 significantly increased in the renal tubular epithelial cells of 18 patients with obstructive nephropathy. Notch3 and mTOR are both involved in autophagy [20,26], but the relationship between Notch3 and mTOR is still unclear. Recent studies have found that the protein expression level of Notch3 was not affected when mTOR was inhibited [13], suggesting that Notch3 may be an upstream regulator of mTOR signaling. Furthermore, the relationship between Notch3 and mTOR was investigated in this study. From our study, co-treatment with the Notch3 inhibitor (DAPT) could reverse the reduction of p-mTOR protein expression, indicating that Notch3 was the major upstream regulator of mTOR. These results are consistent with previous findings by Ivanovska et al. [22]. Besides, the function of Notch3 also was studied. In this study, inhibition of Notch3 activity increased the viability of HG-stimulated MDBK cells, indicating that inhibition of Notch3 expression reduced the injury induced by HG in MDBK cells. Furthermore, results of ultrastructure observation and LC3 immunofluorescence staining showed that Notch3 inhibition could abolish the autophagy induced by HG. Taken together, these findings demonstrated that inhibition of Notch3-mediated mTOR signaling pathway alleviated HG-induced autophagy in MDBK cells.

## 4. Materials and Methods

### 4.1. Cell, Chemical and Reagents

MDBK cells, an immortalized bovine kidney epithelial cell line, were purchased from China Center for Type Culture Collection, Wuhan, China. DMEM Low Glucose medium, DMEM High Glucose medium and DMEM/F12(HAM) medium were purchased from Biological Industries, Kibbutz Beit Haemek, Israel. Notch3 inhibitor (DAPT) was purchased from Selleck, Houston, TX, USA.

### 4.2. Cell Culture

MDBK cells were cultured in DMEM/F12(HAM) medium containing 10% fetal bovine serum (Gibco Life Technologies, Grand Island, NY, USA), 100 U/mL penicillin, and 100 μg/mL streptomycin (Solarbio, Beijing, China). MDBK cells were incubated at 37 °C and 5% CO_2_. When the density of MDBK cells reached 50–60% in a T25 cell bottle, MDBK cells were cultured and incubated at 37 °C and 5% CO_2_ in DMEM Low Glucose medium containing different concentrations of glucose (5.5, 10.5, 15.5, and 25.5 mM), respectively.

### 4.3. Cell Counting Kit-8 (CCK-8) Assay for Cell Growth

Trypsinized MDBK cells (2 × 10^5^ cells) were resuspended in complete DMEM/F12(HAM) medium and seeded in a 96-well plate and then incubated with 5.5, 10.5, 15.5, and 25.5 mM glucose in the previously mentioned DMEM Low Glucose medium under 37 °C, 5% CO_2_. Cell proliferation was evaluated by CCK-8 assay following the manufacturer’s instructions (Solarbio) after 24 h. At least triplicate repeats were completed in all the experiments.

### 4.4. Quantitative Real-Time PCR (qRT-PCR) Analysis

Primers for qRT-PCR were designed by Primer Premier Software 5.0 (Premier Biosoft International, San Francisco, CA, USA). The sequences are shown in Table 1. The qRT-PCR was performed in a 20 μL reaction per well in a 96-well plate containing 3 μL diluted cDNA, 10 μL SYBR^®^ Premix Ex Taq II (Tli RNASEH Plus), 0.4 μL each upstream and downstream primers (10 μM), and 6.2 μL ddH2O. The qRT-PCR amplification procedure was as follows: 94 °C 30 s; 94 °C 5 s, 60 °C 15 s, 72 °C 10 s, 39 Cycles; Melt Curve: 65 °C→95 °C. The qRT-PCR reactions were performed on the CFX96 Quantitative Real-time PCR system (Bio-Rad) by using a qRT-PCR Kit (Takara Bio, San Francisco, CA, USA). Melting curve analysis verified the reliability of each qRT-PCR reaction. Quantitative measurements were determined by using the 2^−ΔΔCt^ method, and the mRNA expression of the β-actin gene was used as the internal control.

### 4.5. Western Blot Analysis

MDBK cells were lysed by a radioimmunoprecipitation assay (RIPA, Solarbio) lysis buffer with phenylmethylsulfonyl fluoride (PMSF, MCE, Monmouth Junction, NJ, USA). The concentration of proteins was measured using a bicinchoninic acid (BCA, Thermo Fisher Scientific, Waltham, MA, USA) protein assay kit. The proteins were separated by SDS polyacrylamide gel electrophoresis (SDS-PAGE, Thermo Fisher Scientific) and were transferred to polyvinylidenedifluoride (PVDF, Thermo Fisher Scientific) membranes. The membranes were blocked in a nonfat dry milk solution for 30 min. Then, the membranes were incubated overnight at 4 °C with Notch3 (Affinity, Cincinnati, OH, USA, DF7193, 1:1000 dilution), anti-mTOR (Bioss, Beijing, China, bs-1992R, 1:1000 dilution), anti-p-mTOR (Affinity, AF3308, 1:1000 dilution), anti-LC3A (Novus Biologicals, Centennial, CO, USA, NB100-2331, 1:1000 dilution), anti-p62 (GeneTex, Irvine, CA, USA, GTX100685, 1:1000 dilution) and anti-Beclin1 (CST, Boston, MA, USA, 3495T, 1:1000 dilution) antibody primary antibodies in dilution buffer. After washing with Tris-buffered saline Tween-20 (Solarbio), the membranes were incubated with horseradish peroxidase (HRP)-conjugated anti-rabbit (Solarbio, SE134, 1:3000 dilution) at room temperature for 30 min. The membranes were developed using the ECL Western blot system (Tanon, Shanghai, China) according to the manufacturer’s instructions.

### 4.6. Immunofluorescence Analysis

MDBK cells were seeded in 24 mm glass-bottomed microwell dishes. After was cultured according to the description mentioned above, MDBK cells were then washed with PBS three times and fixed with 4% paraformaldehyde (Servicebio, Wuhan, China) for 15 min. Next, 0.1% TritonX-100 (Servicebio) was used for cell permeabilization for 20 min. The cells were blocked with 3% BSA (Servicebio) in PBS at room temperature for 30 min and then were incubated with anti-LC3A (Novus Biologicals, NB100-2331, 1:100) overnight at 4 °C. The next day, the cells were incubated with CY3 (Servicebio, GB21303, 1:300) at room temperature for 1 h. The 4,6-diamidino-2-phenylindole (DAPI, Servicebio) counterstain was used to show the MDBK cells’ nuclei. Finally, the stained MDBK cells were observed by immunofluorescence microscopy (Nikon, Tokyo, Japan, Tanon-5200).

### 4.7. Transmission Electron Microscopy (TEM) Analysis

MDBK cells were cultured as described above. MDBK cells were washed with PBS three times and collected by a cell scraper. Then, the cells were centrifuged at 1000 g for 10 min. Precipitated MDBK cells were fixed with ice-cold 3% glutaraldehyde (Solarbio) and then post-fixed in 1% osmium tetroxide. The cell clumps were cut into ultrathin sections after they were embedded in Epon epoxy resin (Chengdu Rongshengke Biotechnology Co., Ltd., Chengdu, China). The ultrathin sections were stained with 0.1% lead citrate (Chengdu Rongshengke Biotechnology Co., Ltd.) and 10% uranyl acetate (Chengdu Rongshengke Biotechnology Co., Ltd.) and were observed with a transmission electron microscope (JEOL, Beijing, China, JEM-1400PLUS).

### 4.8. Statistical Analyses

All the experiments were performed in triplicates. The data were presented as the mean ± SD and were analyzed using variance SPSS 24.0. The differences in the means were determined by one-way analysis of variance (ANOVA) for multiple comparisons followed by the least significant difference (LSD) test for two-group comparisons among the multiple comparisons. *p*-value of less than 0.05 was considered statistically significant (# or * *p* < 0.05) and *p*-value of less than 0.05 was considered extremely significant (## or ** *p* < 0.01).

## 5. Conclusions

**Figure 9:**
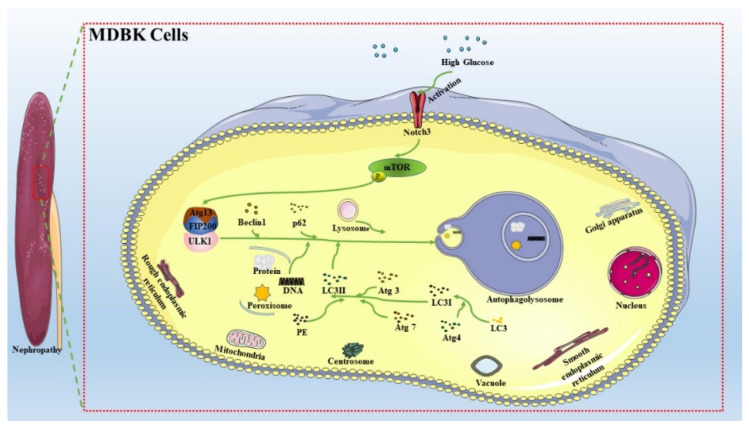
Schematic diagram for the proposed autophagy signaling pathways in MDBK cells regulated by HG. This study provides some theoretical basis for the pathogenesis and treatment of nephropathy induced by hyperglycemia, such as diabetic nephropathy and stress hyperglycemia.

In conclusion, this study demonstrated that the Notch3-mediated mTOR signaling pathway was involved in HG-induced autophagy in MDBK cells, as summarized in Figure 9. This study may provide a certain theoretical basis for the pathogenesis and treatment of nephropathy induced by hyperglycemia, such as diabetic nephropathy and stress hyperglycemia. However, the role of autophagy in HG-induced renal injury needs to be further explored.

## Figures and Tables

**Table 1:** The primers used for Real-time PCR.

Gene Name	Primer	Sequence (5′ and 3′)	Product Length (bp)	Annealing Temperature (°C)	Sequence Number
Notch3	Forward	CAGACACCAATGCCCAGGAC	110	60	XM_003586246.3
	Reverse	CAGGTCTGTAGAGCGGTTCC			
mTOR	Forward	GCTGGCACTTGCTCACAAAA	148	60	XM_002694043.6
	Reverse	GAAGGCATCAATCTTGCGGG			
LC3B	Forward	TGCCGTCCGAGAAAACCTTCAAAC	89	60	XM_027513856.1
	Reverse	CGGGATTTTGGTAGGATGCTGCTC			
p62	Forward	CTGGGAGATGGGCACACC	107	60	XM_024993877.1
	Reverse	TGGGATCTTCCGATGGACCA			
Beclin1	Forward	TCCATTACTTGCCACAGCC	184	60	AM051355.1
	Reverse	GCCATCAGATGCCTCCC			
β-actin	Forward	CGTCCGTGACATCAAGGAGAAGC	143	60	BT030480.1
	Reverse	GGAACCGCTCATTGCCGATGG			

## Data Availability

The data presented in this study are available on request from the corresponding author.

## Supplementary Materials

The following are available online at https://www.mdpi.com/article/10.3390/molecules27103121/s1, Figure S1: The effect of HG on autophagic flux. MDBK cells were pretreated with CQ (10 μM) for 6h, and then stimulated with 25.5 mM glucose for another 24 h, and the protein expression level of LC3 was detected by the Western bolt. ** *p* < 0.01 compared with control group.

## Sample Availability

Samples of the compounds are not available from the authors.
