# Supplementary material for: Notch3-Mediated mTOR Signaling Pathway Is Involved in High Glucose-Induced Autophagy in Bovine Kidney Epithelial Cells
Source: Molecules. 2022 May 13;27(10):3121. doi: 10.3390/molecules27103121 (PMC9143202; doi:10.3390/molecules27103121)
Supplement: Supplementary file 1 [file molecules-27-03121-s001.zip › molecules-1671987-supplementary.pdf]

molecules

Article

Notch3-Mediated mTOR Signaling Pathway Is Involved in High Glucose-Induced Autophagy in Bovine Kidney Epithelial Cells

Yaocheng Cui [1,†], Jing Fang [1,†], Hongrui Guo [1], Hengmin Cui [1], Junliang Deng [1], Shumin Yu [1], Liping Gou [1], Fengyuan Wang [2], Xiaoping Ma [1], Zhihua Ren [1], Yue Xie [1], Yi Geng [1], Ya Wang [1] and Zhicai Zuo [1,*]

[1] Key Laboratory of Animal Disease and Human Health of Sichuan Province, College of Veterinary Medicine, Sichuan Agricultural University, Chengdu 611130, China; cuiyaocheng@stu.sicau.edu.cn (Y.C.); fangjing4109@163.com (J.F.); guohongrui@sicau.edu.cn (H.G.); cuihengmin2008@sina.com (H.C.); dengjl213@126.com (J.D.); yayushumin@163.com (S.Y.); glping0827@163.com (L.G.); mxp886@sina.com (X.M.); zhihua_ren@126.com (Z.R.); zhandegaokandey123@163.com (Y.X.); gengyisicau@126.com (Y.G.); wangyayang@126.com (Y.W.)

[2] College of Animal & Veterinary Sciences, Southwest Minzu University, Chengdu 610041, China; wfy_sccd@163.com

* Correspondence: zzcjl@126.com; Tel.: +86-180-3064-8320

† These authors contributed equally to this work.

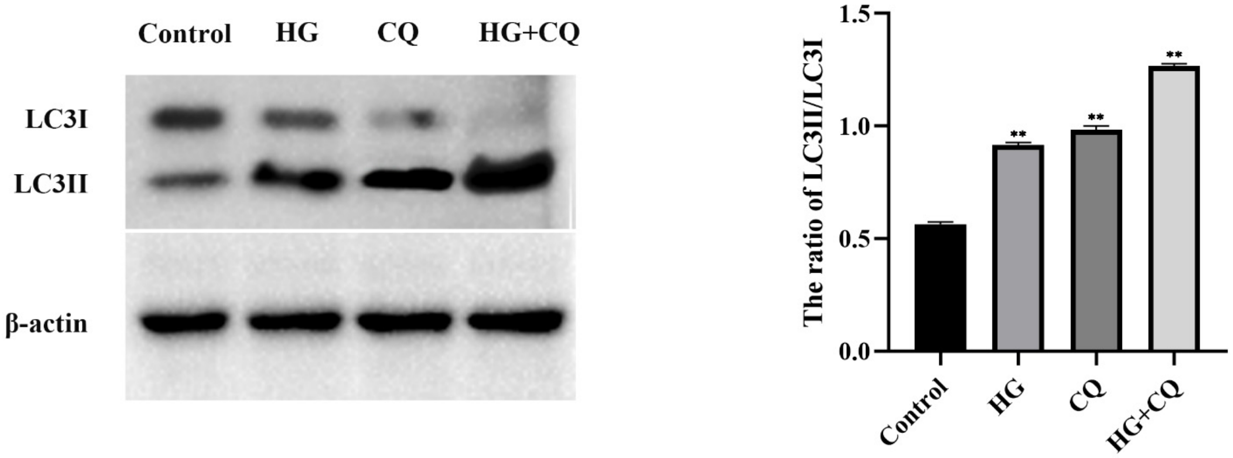

Figure S1. The effect of HG on autophagic flux. MDBK cells were pretreated with CQ (10 μM) for 6h, and then stimulated with 25.5 mM glucose for another 24 h, and the protein expression level of LC3 was detected by the Western bolt. ** $p < 0.01$ compared with control group.
